# Research on Internal Force Detection Method of Steel Bar in Elastic and Yielding Stage Based on Metal Magnetic Memory

**DOI:** 10.3390/ma12071167

**Published:** 2019-04-10

**Authors:** Caoyuan Pang, Jianting Zhou, Ruiqiang Zhao, Hu Ma, Yi Zhou

**Affiliations:** 1 College of Civil Engineering, Chongqing Jiaotong University, Chongqing 400074, China; 622160086030@mails.cqjtu.edu.cn; 2 College of Materials Science and Engineering and Engineering Research Center of Bridge Structure and Material in the Mountainous Area of Ministry of Education, Chongqing Jiaotong University, Chongqing 400074, China; rqzhao@cqjtu.edu.cn; 3 Chongqing Rail Transit (Group) Co., Ltd., Chongqing 401120, China; cqmetromh@163.com; 4 Chongqing Yapai Bridge Engineering Quality Inspection Co., Ltd., Chongqing 401120, China; boatzy@163.com

**Keywords:** metal magnetic memory, steel bar, internal tensile force detection, elastic stage, yielding stage

## Abstract

Based on the metal magnetic memory effect, this paper proposed a new non-destructive testing method for the internal tensile force detection of steel bars by analyzing the self-magnetic flux leakage (SMFL) signals. The variation of the SMFL signal of the steel bar with the tensile force indicates that the curve of the SMFL signal has a significant extreme point when the tensile force reaches about 65% of the yield tension, of which the first derivative curve has extreme points in the elastic and yielding stages, respectively. To study the variation of SMFL signal with the axial position of the steel bar under different tensile forces, a parameter reflecting the fluctuation of the SMFL signal along the steel bar is proposed. The linear relationship between this parameter and the tensile force can be used to quantitatively calculate the tensile force of steel bar. The method in this paper provides significant application prospects for the internal force detection of steel bar in the actual engineering.

## 1. Introduction

The existing detection technology cannot directly measure the internal stress of the steel bar, but indirectly estimates the magnitude and direction of the stress by measuring the variation of certain material parameters during the stress process [1]. The steel bars in the reinforced concrete structure are mainly used to assist the concrete to resist tensile force. From loading to the break, the four stages of elasticity, yielding, strengthening, and necking are generally experienced. When the internal tensile force reaches the yield point, the plastic deformation of the steel bar will cause the reinforced concrete structure to undergo excessive deformation and excessive cracking, which mean the structure cannot be used normally as a result. Therefore, it is critical to effectively identify whether the tensile force of the steel bar reaches the yield stage and accurately measure the internal tensile force during the tension process for the structure safety assessment in the actual engineering.

For the stress measurement in steel components, destructive detection often uses a method of semi-damage or micro-damage to release the internal stress, which changes the local stress of structure by cutting, grooving, and collar drilling [2,3]. The traditional non-destructive detection methods of steel stress are mainly ultrasonic testing [4,5], X-ray detection [6], eddy current testing [7,8], infrared thermal imaging [9], and acoustic emission technology [10], etc. However, these traditional detection methods have certain deficiencies. Ultrasonic testing requires acoustic coupling agents as well as surface treatment. The X-ray method has radioactivity, the method can only detect the surface residual stress of the structure, and the detection equipment is expensive and time consuming [11]. Eddy current testing is mainly used for non-ferromagnetic materials. Thermal imaging methods (infrared, eddy current, microwave excitation) have the detection of small depth and are not suitable for use in reinforced concrete structures due to the presence of a protective layer [9]. Additionally, these traditional non-destructive testing methods cannot distinguish the stress concentration and the mapping relationship between the stress distribution state and the damage causes [12].

Steel bar is one kind of typical ferromagnetic material, of which the microstructure can be considered to consist of many magnetic domain arrangements [13]. When an external force acts on the ferromagnetic material, it will cause a change in the internal magnetic domain structure, and the shape and dimension of the internal ferromagnetic crystal will be changed. The macroscopic performance from the influence is the change of the magnetic properties. Conversely, if the ferromagnetic material is subjected to an external magnetic field, the dimensions and shape of the internal ferromagnetic crystal will also be changed, which is called the piezomagnetic effect [14,15]. The magnetic nondestructive testing method of ferromagnetic materials in the actual engineering is based on this effect, of which the typical methods include magnetic anisotropy technology [16], Barkhausen magnetic noise technology [17], and metal magnetic memory (MMM) technology. The basic principle of the MMM non-destructive testing technology can be summarized as follows: when the ferromagnetic material is subjected to external force or internal damage, under the action of the earth magnetic field, the internal magnetic domain of the material is irreversibly redirected, and the external MMM signal will be changed [18,19]. Therefore, according to the theory of MMM, a relationship can be established between the internal stress of the ferromagnetic material and the surface MMM signal, and accordingly, the internal force of the structure can be obtained by collecting the MMM signal above the steel structure surface in the actual internal stress detection [20]. The theory was first proposed by Russian expert Doubov [19] and has been developed in theory and application for more than two decades. Compared with other magnetic methods [21,22] and traditional non-destructive testing methods, MMM testing technology can be used to detect early defects and damage sites of ferromagnetic materials. Strong magnetic field excitation is not required for magnetization of ferromagnetic metal materials, and the equipment for collecting MMM signals is simple and low in cost [23]. The MMM employs the earth magnetic field as the stimulus source instead of a strong artificial magnetic field, so the magnetic signals are also called as self-magnetic flux leakage (SMFL) signals. The variation of SMFL signal is related to many factors such as the initial magnetic field, microstructure, chemical composition, and shape and size of the ferromagnetic materials [24], and lots of scholars around the world have done a lot of researches on this [25]. On the research of relationship between internal stress of ferromagnetic material and SMFL signal, Zhou [26] studied the relationship between the bearing capacity of reinforced concrete beams after corrosion and SMFL signals and proposed a gradient K to determine the degree of corrosion. Ren [25] studied the relationship between SMFL signals and stress in different stress stages, and derived related calculation formulas accordingly by the plain carbon structural steel 45 loading test. Dong [27] et al. have tested the Q235 steel, and concluded that the variation of the normal component of stress-induced stray filed can reflect the different deformation stages of static tensile test during the loading process. When using the MMM method to detect the stress of the steel bar, it is only necessary to collect the SMFL on its surface, and it is not necessary to perform magnetization or other processing on the steel bar before detection. The SMFL acquisition equipment is simple and rather portable [23], as long as the SMFL signal is formed under the action of the geomagnetic magnetic field, rust, stress, and so on. It is relatively stable only if it is not subject to too much interference from the outside, and is not affected by factors such as temperature changes. On the other hand, the reinforced concrete structure in civil engineering usually has a certain thickness of protective layer on the surface of the steel bar. The MMM method does not need to peel off the concrete, and the detection sensor does not need to touch the steel bar; the concrete has almost no influence on the test results either.

In order to study the variation law of the SMFL signal on the surface of the steel bar with its internal tensile force and propose the tensile force detection and calculation method of the steel bar in the elastic and yielding stage, the HRB400 threaded steel bars (TSB) that are commonly used in civil engineering are taken as the test specimens. Through the static loading by the electric universal testing machine (EUTM, DNS300, Changchun Research Institute for Mechanical Science Co., Ltd, Changchun, China), relevant tests were carried out for the TSBs of different diameters, and the analysis and discussion were conducted according to the test results.

## 2. Materials and Methods 

**Figure 1:**
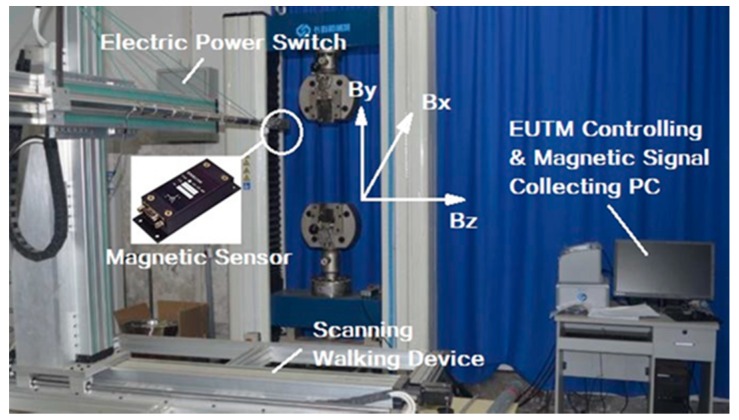
Electric universal testing machine (EUTM) and automatic triaxial self-magnetic flux leakage (SMFL) signal acquisition device.

The length of the TSB specimen used in this test is 500 mm, and its chemical composition is shown in Table 1. A total of five diameter TSBs were used, which were 12 mm, 16 mm, 18 mm, 20 mm, and 25 mm (Ф 12, Ф 16, Ф 18, Ф 20, and Ф 25), and three test pieces were prepared for each diameter, numbered 1#, 2#, and 3#, respectively. The test instrument used for tensile loading of TSBs is EUTM. At the same time, the three-axis SMFL signal acquisition device designed and manufactured by ourselves is used to collect the SMFL signal of TSB during the tensile process. As shown in Figure 1, the device can continuously collect and record the SMFL signals in the X, Y, and Z directions. The magnetic signal sensor of the device is the Honeywell HMR2300 magnetic flux leakage signal collector (Morris Plains, NJ, USA). 

The test consists of two parts. The first part is to study the variation law of the SMFL signal of the TSBs with the tensile force, in which the two ends of 1# TSB in each diameter group are respectively fixed to EUTM, and the static load is stretched under normal temperature conditions. The tensile loading process of 1# TSBs is conducted according to the diameter of the TSB from small to large, and the tensile force starts from 0 kN to the magnitude that breaks the TSB. While measuring the tensile-displacement curve of the 1# TSB, the magnetic sensor is placed above the midpoint of the length of the specimen after the clamp length in the two ends is subtracted from the total length of TSB. The magnetic sensor is used to continuously record the variation of SMFL signal during the entire loading process. Therefore, the first part of the test is also referred to as the fixed-point monitoring test. The second part of the test is to study the variation of the SMFL signal along the axial position of the steel bar under different tensile forces. In this part, the 2# TSB was fixed on the EUTM, and the SMFL signal above the surface of TSB along the axial direction within a certain length was continuously collected under a certain targeted loading displacement (TLD) of EUTM, which is also called that axial scanning test [28,29]. In terms of the EUTM, the two parts of the test mentioned was loaded by the displacement, and the tensile loading rate was 5 mm/min.

**Figure 2:**
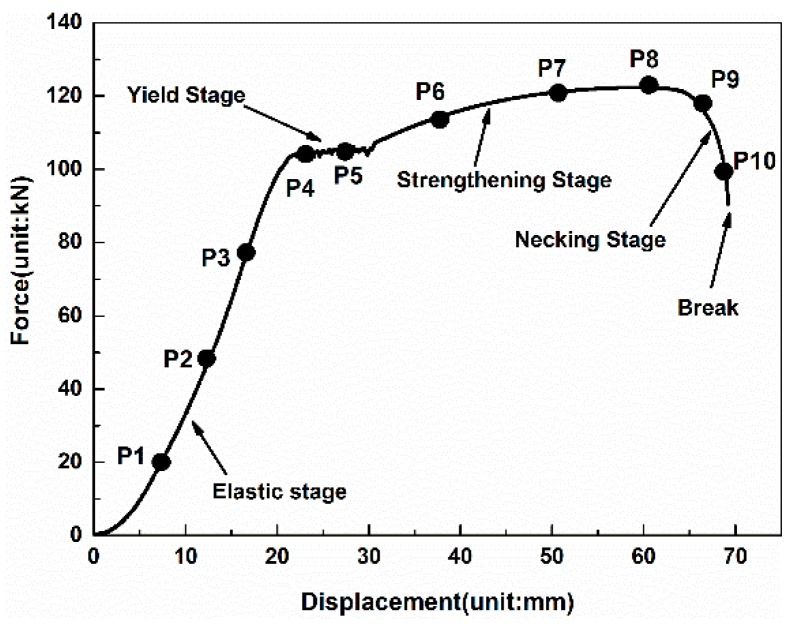
Targeted loading displacement (TLD) points selection indication from the tensile-displacement curve.

The tensile-displacement curve of the 1# TSB measured in the fixed-point monitoring test is used as the basis for the selection of TLDs for the 2# specimen in the axial scanning test. The method is to select 1~3 points for each stage of elasticity, yielding, strengthening, and necking of the 1# specimen from the tensile-displacement curve as the TLDs for the 2# TSB. As shown in Figure 2, taking the 16 mm diameter TSB as an example, points P1–P10 are selected TLDs according to the tensile-displacement curve measured of 1# TSB in this diameter group. In the axial scanning test, the 2# TSB is first loaded to the TLD corresponding to point P1, and then the EUTM is suspended for loading while the scanning test is performing. After the scanning test is finished, the TLD corresponding to the point P2 point is continuously loaded to process the scanning test until the end of test of the TLD corresponding to point P10. In the test, the 3# TSB corresponding to each diameter was used as the spare test piece.

**Figure 3:**
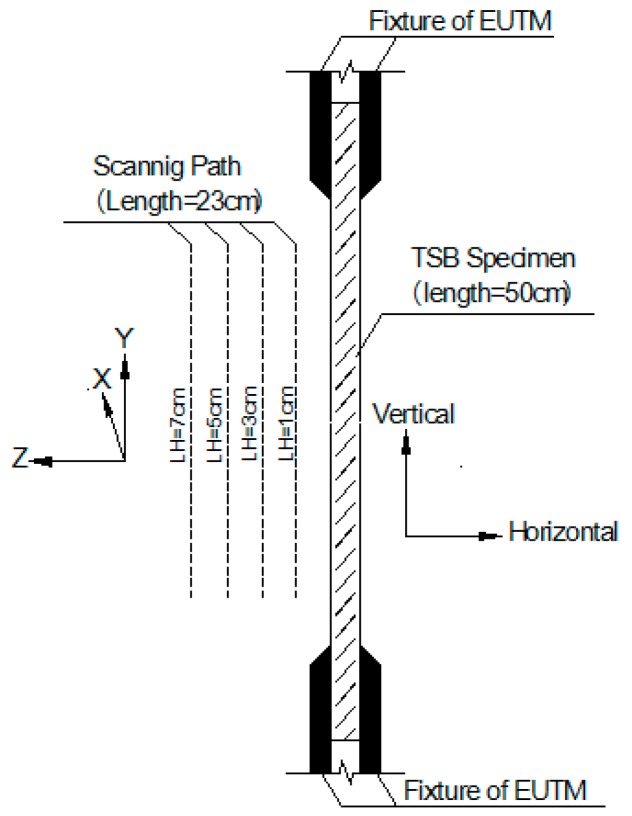
Scanning path diagram of magnetic sensor in the axial scanning test.

In the axial scanning test of 2# TSB, the magnetic sensor of the three-axis SMFL signal acquisition device designed and manufactured by ourselves was scanned along the axial direction of the TSB at four lift-off heights (LHs) paths of 1 cm, 3 cm, 5 cm, and 7 cm, respectively. Because there is a clamping length of the EUTM fixture of about 10 cm at both ends of the TSB, considering that the excessive clamping force will cause local magnetization, the influence cannot be ignored theoretically. Therefore, the axial scanning length is the 230 mm long section in the middle part of TSB after the clamp length in the two ends is subtracted. Figure 3 shows the schematic diagram of the scanning path length and LHs of the magnetic sensor in the axial scanning test.

## 3. Results and Discussion

### 3.1. SMFL Signal Results in the Fixed-Point Monitoring Test

#### 3.1.1. The Variation Law of the Tangential Component (By Signal) of the SMFL Signal

**Figure 4:**
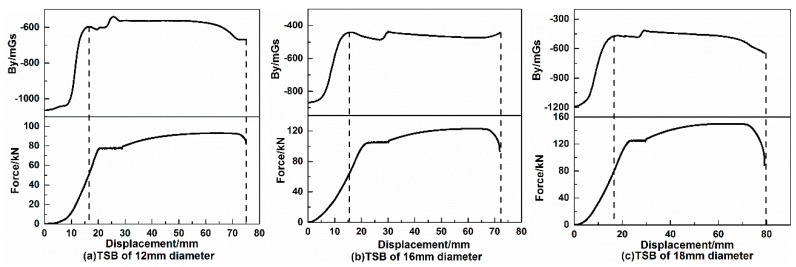
Variation of By signal with displacement during the loading of 1# TSB. (**a**) TSB of 12 mm diameter, (**b**) TSB of 16 mm diameter and (**c**) TSB of 18 mm diameter.

The variation of the SMFL above the surface of the midpoint of TSBs for different diameters during the tensile loading process is plotted, as shown in Figure 4. In order to better study the variation law of SMFL during the loading process of TSBs, the variation curve of the tangential component of SMFL (By signal) with the loading displacement is put together with the tensile-displacement curve of TSB, and the abscissa is the loading displacement of EUTM. Each upper half of Figure 4a–c is the variation curve of the By signal of each diameter TSB with the loading displacement, and each lower part is the tensile-displacement curve corresponding to TSB. Since the results of TSBs were similar for each diameter group, only the results of the TSB with diameters of 12 mm, 16 mm, and 18 mm were shown here. It can be seen from the tensile-displacement curve of each diameter TSB in Figure 4 that the tensile-displacement curve of the TSB is similar to the stress-strain curve of the steel tested in the laboratory, and the TSB corresponding to each diameter still shows four obvious stages in the whole stretching process, namely the elastic stage, the yielding stage, the strengthening stage, and the necking stage. The similarity between the By curve and the tensile-displacement curve can be explained as this: In the initial stage of loading, since the magnetic domain walls inside the TSB can rotate freely and easily, the variation of SMFL signal by the external tensile force is more obvious, so the By signals grows rapidly at this stage. At the latter stage of the loading, due to the increasing internal dislocations and pinning points, the rotation is no longer as free and easy as initially and the change is slow and fluctuates in a small range; as is the tensile-displacement curve, in which the internal force grows rapidly in the elastic stage and varies rather in a small scale after entering the yielding stage. Because the TSBs are all HRB400, the larger the diameter of the TSB, the larger the cross-section surface, and the greater the tension required for the EUTM to stretch the TSB into the yield. During the whole process, as the diameter of the TSBs increases, the corresponding tensile force of the yield stage also becomes larger, as do the loading displacements required for TSB to enter the yielding stage. This is consistent with the theory.

In terms of the variation law of the SMFL of the TSBs in the process of loading, the By signal curve for different TSB diameters is about the same. During the initial period of the loading process, the value of the By signal increases with the loading displacement, of which the numerical value increases largely about 400–700 mGs. When the loading displacement or tensile force reaches a certain size, one extreme point appears on the curve of By signal, and then the By signal enters a “platform stage”, after which it varies in a small range. The ratio of the tensile force corresponding to the extreme point before entering the “platform phase” on the By signal curve to the yield tension is generally close to 65%. In the process from when the By signal enters the “platform stage” to the TSB breaks, the variation range of By signal is small, within 200 mGs.

#### 3.1.2. The Variation Law of the First Derivative of the By Signal

**Figure 5:**
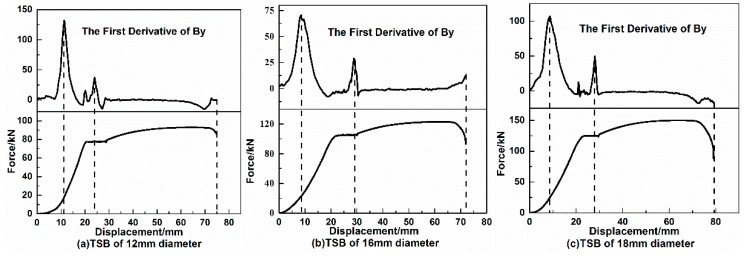
The variation of the first derivative of the By signal with the loading displacement. (**a**) TSB of 12 mm diameter, (**b**) TSB of 16 mm diameter and (**c**) TSB of 18 mm diameter.

In order to study the variation rate of increase and decrease of the By signal during the loading process, the first derivative of the By signal curve corresponding to each diameter of TSB collected during the loading process is taken and plotted, as shown in Figure 5. Each upper half of Figure 5a–c is the first derivative curve of the By signal during the loading process, and each lower half of Figure 5a–c is the tensile-displacement curve. Since the results of each diameter are similar, the TSBs with diameters of 12 mm, 16 mm, and 18 mm are shown here as well. It can be known from Figure 5 that at the beginning of loading, the first derivative of the By signal increases sharply, and then reaches a fairly large peak. At this peak, the growth rate of the By signal reaches the maximum value; that is, the stagnation point during the initial loading stage on the By signal curve in Figure 4. Then, the TSB enters the yielding stage. In this stage, the first derivative curve also has a relatively obvious peak, but compared with the peak value at the beginning of loading, the value at this time is one-third to one-half of that. The value of other parts on the first derivative curve of By except for the two obvious peaks are close to 0, which indicates that the growth rate of these parts is relatively stable and does not change too much. At the moment when the TSB was loaded to break, the curve of the first derivative of the By signal did not change significantly. For the first derivative curve of the By signal, the quite obvious features are summarized as the two peaks appearing in the elastic stage and the yield stage, respectively. The peak value appearing in the elastic stage is several times that of the peak appearing in the yielding stage. The peaks appearing on the first derivative curve of the By signal in the yield stage are located in the second half of the yielding stage. All TSBs have peaks greater than 50 and less than 150 in the elastic phase, and the peaks in the yielding stage are less than 50 but greater than 20.

In the fixed-point monitoring test, the reason for the above phenomena can be explained as follows. TSB is a typical ferromagnetic material, and the magnetic domain is the microscopic component of its internal structure. When an external force acts on TSB, it will cause an increase in the internal tensile force of TSB. According to the piezomagnetic effect, internal magnetic domain of the TSB rotates directionally under the internal forces of TSB, and the direction of rotation is the same as the tensile force, so the magnetization in the direction of the tensile force is enhanced, resulting in the SMFL signal increases rapidly. This coincides with the quick increase in the By signal at the beginning of loading and the occurrence of stagnation on the By signal curve in Figure 4. When the tensile force reaches a certain value, the dislocations increase rapidly, resulting in excessive dislocation density, increased pinning points, and limited directional rotation of the magnetic domain wall [30]. The magnetization is basically no longer increased, showing a small change in the amplitude of the SMFL signal, which can explain the stage of the platform appearing on the By signal curve during loading, theoretically. As the tensile force increases further, excessive accumulation of the dislocation will destroy the directional arrangement of the original magnetic domains, causing the magnetization and the SMFL signal of TSB to decrease slowly [31,32,33], of which this phenomenon is usually manifested in the later stages of the loading process. Therefore, in the experiment, after the By signal curve enters the platform stage, the variation of By signal is small, and the curve shows a relatively stable shape.

### 3.2. The Results of By Signal in the Axial Scanning Test

**Figure 6:**
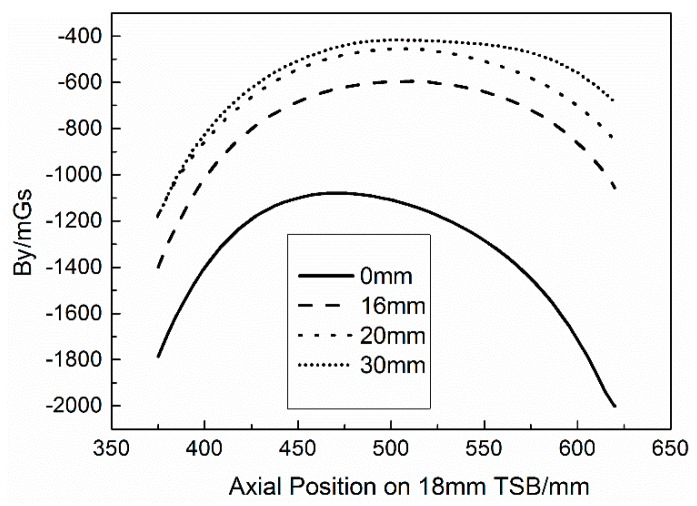
Axial scanning test results of some TLDs of 18 mm TSB at LH = 3 cm.

Figure 6 shows the axial scanning test results of a 230 mm length section of the 18 mm diameter TSB with the TLDs of 4 mm, 16 mm, 20 mm, and 30 mm when the LH value is 3 cm. Because the TLDs of the axial scanning test result are too many, in order to more clearly show the By signal variation along the axial direction of TSB, only the results of the above TLDs are shown in the figure. It can be seen from Figure 6 that for the distribution of the axial scanning test results along the axial direction of TSB, the By signal in the two ends is small and in the middle section of specimen is large, of which the minimum value on the entire TSB scanning curve appears at one of the two ends, and the maximum value appears in the middle, but not necessarily at the midpoint of the curve. The By signal values are continuously and evenly transitioned from the ends to the middle without significant numerical jump. The whole axial By scanning curve is quite smooth and there is no fluctuation on it locally. According to the knowledge of MMM, under the action of the geomagnetic magnet field, the phenomenon of spontaneous magnetization of metal ferromagnetic work pieces occurs. In the defect of the work piece, the leakage magnetic field will be generated, and the abnormal change of the magnetic field will occur. The position of non-defective will show a stable and consistent magnetic field distribution in contrast [34,35]. The TSB used for the axial scanning test has no local defects. During the loading process, the internal stress of TSB is theoretically the same when the TLD is kept in one value. Therefore, when the TSB is subjected to magnetization caused by the interaction of the geomagnetic magnetic field and internal stress, the result of the axial scanning test displays the smooth curve. The reason the signal value at both ends of the scanning section is smaller than the middle can be explained as the end effect of TSB. Additionally, as the TLD value increases, the whole scanning curve has a tendency to move upwards; that is, the overall value increases. At the beginning of the loading process, the increment of the overall upward movement of the curve is large while the increment of the upward movement is small at the latter loading process, of which the variation at one point on the axial scanning curve of the By signal is similar to the test results in the fixed-point monitoring test.

Considering that in the actual engineering, the detection and monitoring of TSB differ in time continuity, and the magnitude of the increments at the ends and the middle section are usually different. Furthermore, it is impossible to determine the stress stage of the TSB based on the absolute value of the By signal in the curve detected in the axial scanning test, because of the many complicated factors such as the geomagnetic field, the environmental magnetic field, the chemical composition, and the internal stress. Even if the internal stresses of TSBs are the same, the absolute values of the By signals may be different. In order to quantitatively detect the tensile force of the TSB, according to the amplitude of the By signal along the axial direction under different TLDs, the “force-induced magnetic fluctuation parameter” can be defined, which is given by Equation (1):

(1) $$A_{T}=\ln(\frac{{\mathrm{By}}_{\max}-{\mathrm{By}}_{\min}}{b_{y}})$$

where ${\mathrm{By}}_{\max}$ is the largest value on the axial scanning test curve of By, ${\mathrm{By}}_{\min}$ is the minimum value on the curve, and $b_{y}$ is the unit vector of the SMFL signal of the TSB along the y-axis direction, of which size is 1 mGs. The larger the value of $A_{T}$, the greater the fluctuation of the By signal in the axial direction.

**Figure 7:**
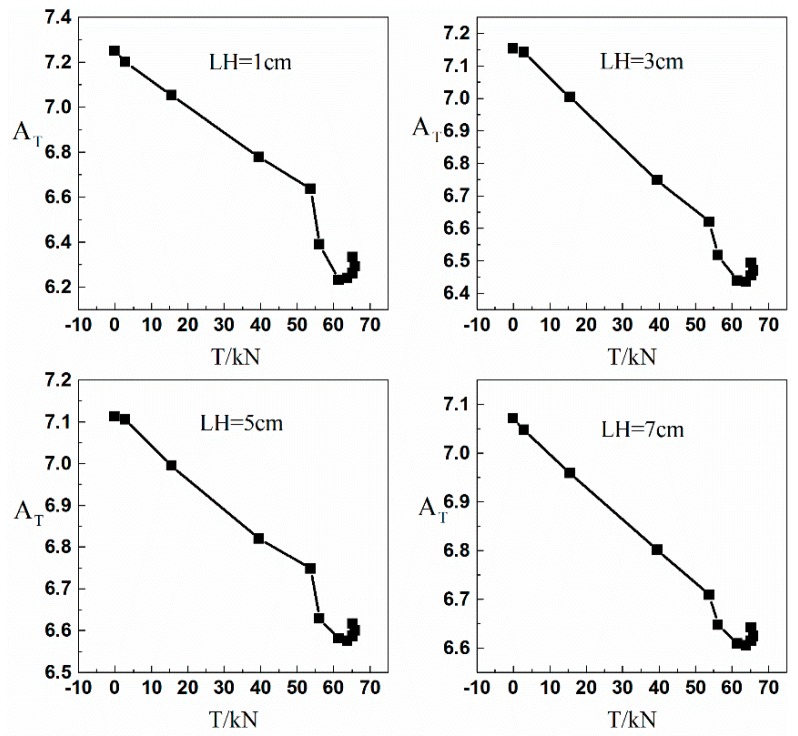
$A_{T}$-T diagram of the By signal of 12 mm diameter TSB.

Table A1, Table A2, Table A3, Table A4, Table A5, Table A6, Table A7, Table A8, Table A9, Table A10, Table A11 and Table A12 in the Appendix A of this paper show the $A_{T}$ calculation results of 12 mm, 20 mm, and 25 mm TSB at LH of 1 cm, 3 cm, 5 cm, and 7 cm, respectively. Because the results are similar, only the calculation results of the above diameters are listed. The calculated $A_{T}$ is taken as the ordinate, the corresponding tensile force value of each TLD on the tensile-displacement curve is taken as the abscissa, and the $A_{T}$-T graphs of TSBs of different diameters under different LHs can be drawn, as shown in Figure 7.

**Figure 8:**
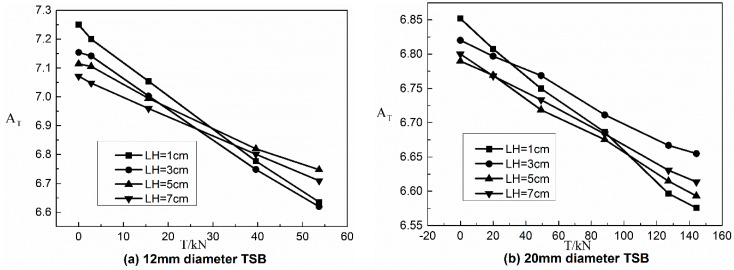

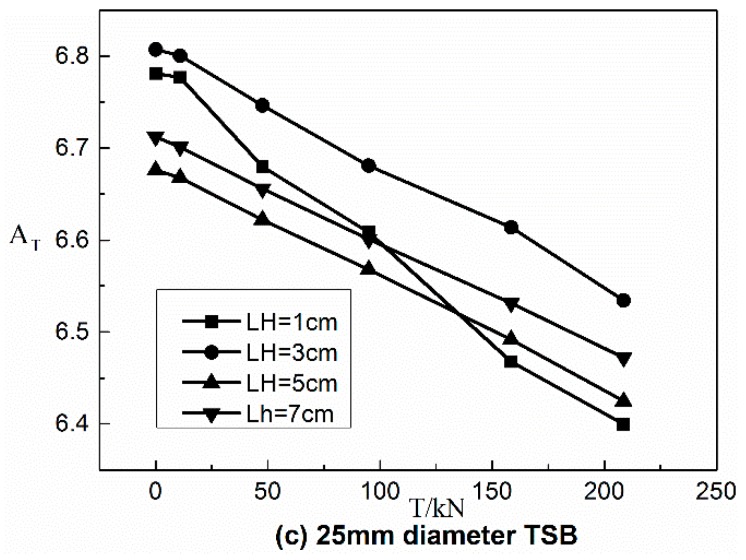
The linear section of A_T_-T graphs under different LHs for different TSB diameters. (**a**) 12 mm diameter TSB; (**b**) 20 mm diameter TSB; (**c**) 25 mm diameter TSB.

Figure 7 is $A_{T}$-T graph of the 12 mm TSB at LH of 1 cm, 3 cm, 5 cm, and 7 cm, respectively. Since the $A_{T}$-T graph show the same regular law of linear relationship between $A_{T}$ and T, only the 12 mm TSB $A_{T}$-T graph is shown here. From the graph, it can be concluded that for each diameter TSB under any one LH condition in the axial scanning test, as the tensile force increases $A_{T}$ decreases with the increase of T in the linear elastic range of the TSB. For each diameter of TSB, there is a linear relationship between $A_{T}$ and T in the range of linear elastic stage. Outside the linear elastic stage, some $A_{\mathrm{TS}}$ decreases with the increase of T, and some $A_{\mathrm{TS}}$ increases with the increase of T, in which there is no obvious relationship between the two. This phenomenon can be explained by the fact that at the initial loading stage, the dislocations and pinning points accumulated inside the ferromagnetic material tend to be less, and the magnetic domains can be freely oriented while the direction of rotation is the same as the direction of the tensile force, so the SMFL signal variation law is stable. When the loading enters a certain stage, the amount of internal dislocations and pinning points of the ferromagnetic material is enough to limit the free rotation of the magnetic domain. At this time, the variation law of the SMFL is not obvious. Drawing of the linear section of $A_{T}$-T graphs for the same diameter TSB under different LHs in one coordinate system by diameter of TSB is shown in Figure 8.

Figure 8 shows the linear section of the $A_{T}$-T graph of TSBs for different diameters at different LHs. In the linear section of all $A_{T}$-T diagrams in Figure 8, the variation trend of $A_{T}$ decreases with increasing T, and the linear relationship between the two is excellent. For the TSBs of different diameters, the overall absolute value of $A_{T}$ shows a decreasing trend with the increase of the diameter of TSB. For the same diameter TSB, the linear slope shows an increasing trend as the axial scanning LH increases. In combination with the slope of the linear segment in the $A_{T}$-T diagram for the By signal measured in the test, the tensile force T of the steel bar can be calculated within the range of linear elasticity stage, just as follows:

(2) $$A_{T}=\ln(\frac{{\mathrm{By}}_{\max}-{\mathrm{By}}_{\min}}{b_{y}})$$

(3) $$A_{T}=K\cdot T+C$$

(4) $$\ln(\frac{{\mathrm{By}}_{\max}-{\mathrm{By}}_{\min}}{b_{y}})=K\cdot T+C$$

(5) $$T=\frac{\ln(\frac{{\mathrm{By}}_{\max}{-\mathrm{By}}_{\min}}{b_{y}})-C}{K}$$

where ${\mathrm{By}}_{\max}$ and ${\mathrm{By}}_{\min}$ can be obtained by scanning the entire steel bar in the actual engineering test, K and C are the slope and longitudinal intercept of the linear segment in the $A_{T}$-T diagram, which can be determined according to the test in advance.

### 3.3. Influence of TSB Diameter on the Test Results

**Figure 9:**
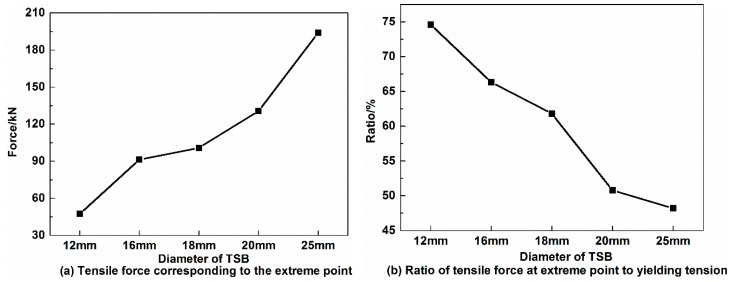
The tensile force corresponding to the extreme point on the By curve in the fixed-point monitoring test with different TSB diameters. (**a**) Tensile force corresponding to the extreme point, (**b**) Ratio of tensile force at extreme point to yielding tension.

As shown in Figure 9a, in the fixed-point monitoring test, the tensile force corresponding to the extreme point before entering the “platform stage” on the By signal curve is 49.8 kN for the 12 mm TSB, 67.7 kN for the 16 mm TSB, 80.1 kN for the 18 mm TSB, 91.2 kN for the 20 mm TSB, and 136.7 kN for the 25 mm TSB, which denotes that the magnitude of the tensile force corresponding to the extreme point increases as the diameter of TSB increases. The ratio of this tensile force to that corresponding to the yield tension is 64.68% for the 12 mm TSB, 64.48% for the 16 mm TSB, 64.08% for the 18 mm TSB, 64.01% for the 20 mm TSB, and 63.89% for the 25 mm TSB, which decreases slightly as the diameter of TSB increases, as shown in Figure 9b.

**Figure 10:**
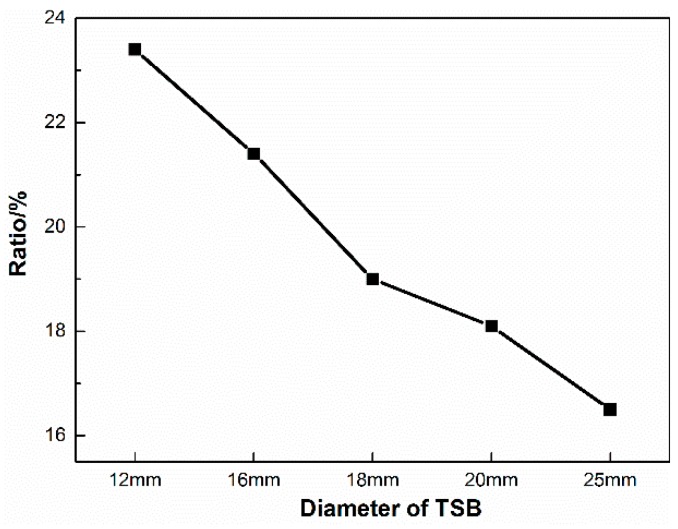
The ratio of the tensile force corresponding to the peak value on the first derivative curve of the By signal to the yield tension with different TSB diameters of the elastic stage in the fixed-point monitoring test (%).

As shown in Figure 10, in terms of the tensile force corresponding to the peak in the elastic stage on the first derivative curve of the By signal, 18.0 kN is for the 12 mm TSB, and the ratio of which to the yield tension is 23.4%, 22.5 kN and 21.4% for the 16 mm TSB, 23.8 kN and 19.0% for the 18 mm TSB, 25.7 kN and 18.1% for the 20 mm TSB, and 35.3 kN and 16.5% for the 25 mm TSB. The ratio decreases with the increase of the TSB diameter.

**Figure 11:**
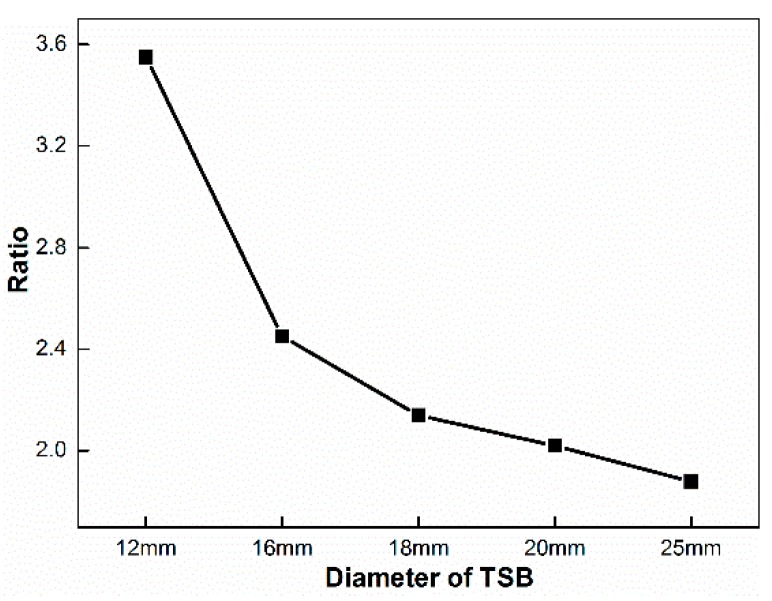
The ratio of the two peak values on the first derivative curve of the By signal in the fixed-point monitoring test with different TSB diameters.

In the fixed-point monitoring test, in terms of the ratio of magnitudes at peaks in the two stages on the first derivative curve of By signal, 3.55 is the ratio of 12 mm TSB, 2.45 is the ratio of the 16 mm TSB, 2.14 for the 18 mm TSB, 2.02 for the 20 mm TSB, and 1.88 for the 25 mm TSB, which indicates that the ratio decreases as the diameter of TSB increases, as shown in Figure 11.

**Figure 12:**
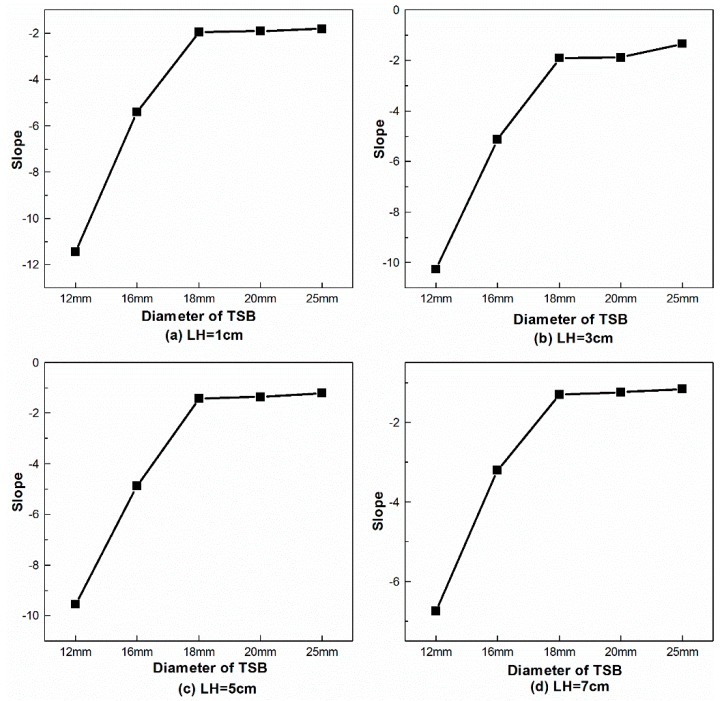
The slope of the linear segment in the $A_{T}$-T diagram with different TSB diameters in the axial scanning test. (**a**) LH = 1 cm, (**b**) LH = 3 cm, (**c**) LH = 5 cm and (**d**) LH = 7 cm.

Figure 12 shows the slope of the linear segment in the $A_{T}$-T diagram in the axial scanning test, which signifies that the slope increases as the diameter increases at the same LH.

## 4. Conclusions

In order to study the variation law of the SMFL signal above the surface of the steel bar with its internal tensile force and propose the tensile force detection and calculation method of the steel bar in the elastic and yielding stage, the fixed-point monitoring test and the axial scanning test under different working conditions are carried out, and the following conclusions can be drawn:

(1) In terms of the variation of the SMFL signal with the tensile force, the tangential component of SMFL (the By signal) is initially increased with the increase of the loading displacement. When the tensile force is close to, but slightly less than, 65% of the yield tension, the extreme point appears on the By signal curve, and then the By signal enters the “platform stage” with a small range of fluctuating following. The first derivative curve of the By signal has a large numerical peak at the elastic and yielding stages, respectively.
(2) This paper proposes the “force-induced magnetic fluctuation parameter” $A_{T}$ that reflects the fluctuation amplitude of the By signal along the axial direction of TSB. In the linear elastic stage, there is a linear relationship between $A_{T}$ and the internal tensile force of TSB, of which the slope is constant at the same LH, and the tensile force of TSB can be calculated by this way.
(3) In the fixed-point monitoring test, the amount of tensile force corresponding to the extreme point on the By signal curve before entering the “platform stage” increases as the diameter of TSB increases, and the ratio between this tensile force and the yield tension decreases as the diameter of TSB increases. The ratio of magnitudes at peaks in the two stages on the first derivative curve of By signal decreases as the diameter of TSB increases. The slope of the linear segment in the $A_{T}$-T diagram in the axial scanning test increases as the TSB diameter increases at the same LH.
(4) The method in this paper provides significant application prospects for the internal force detection of steel bar in actual engineering, which has low cost, simple equipment, and easy operation.

## Figures and Tables

**Table 1:** Chemical composition mass fraction of threaded steel bars (TSB) (%).

Type of Steel	Chemical Composition				
	C	Si	Mn	P	S
HRB400	0.2	0.4	1.3	0.03	0.02

## Appendix A

**Table A1:** Calculation result of $A_{T}$ for 12 mm diameter steel bar (LH = 1 cm).

	TLD	0	4	8	12	16	20	30	40	50	60	70
Parameter												
${\mathrm{By}}_{\max}$		−529.5	−530.7	−536.2	−502.3	−495.3	−454.7	−490.5	−482.1	−470.9	−457.8	−433.9
${\mathrm{By}}_{\min}$		−1937.7	−1870	−1693.5	−1379.7	−1256.9	−1049.7	−999.1	−994.1	−994.7	−997.3	−997.5
T		0	2.8	15.6	39.6	53.7	56.1	61.5	63.8	65.3	65.9	65.2
$A_{T}$		7.2501	7.1999	7.0538	6.7770	6.6354	6.3886	6.2317	6.2383	6.2611	6.2906	6.3343

**Table A2:** Calculation result of $A_{T}$ for 12 mm diameter steel bar (LH = 3 cm).

	TLD	0	4	8	12	16	20	30	40	50	60	70
Parameter												
${\mathrm{By}}_{\max}$		−668.7	−663	−654	−571.3	−584.9	−581.6	−555	−543.1	−528.4	−514.7	−495.5
${\mathrm{By}}_{\min}$		−1947.9	−1926.6	−1754	−1423.5	−1334.3	−1258.3	−1180.5	−1166.1	−1163.7	−1159.9	−1157.1
T		0	2.8	15.6	39.6	53.7	56.1	61.5	63.8	65.3	65.9	65.2
$A_{T}$		7.1540	7.1417	7.0031	6.7478	6.6193	6.5172	6.4386	6.4345	6.4541	6.4696	6.4947

**Table A3:** Calculation result of $A_{T}$ for 12 mm diameter steel bar (LH = 5 cm).

	TLD	0	4	8	12	16	20	30	40	50	60	70
Parameter												
${\mathrm{By}}_{\max}$		−761.5	−751.3	−732.5	−645.4	−647.8	−644.4	−605.1	−589.3	−574	−558.5	−540.9
${\mathrm{By}}_{\min}$		−1990.5	−1969.9	−1823.1	−1561.3	−1499.9	−1400.7	−1326.5	−1306.1	−1299.5	−1293.7	−1288.7
T		0	2.8	15.6	39.6	53.7	56.1	61.5	63.8	65.3	65.9	65.2
$A_{T}$		7.1140	7.1055	6.9945	6.8199	6.7477	6.6284	6.5812	6.5748	6.5869	6.6001	6.6171

**Table A4:** Calculation result of $A_{T}$ for 12 mm diameter steel bar (LH = 7 cm).

	TLD	0	4	8	12	16	20	30	40	50	60	70
Parameter												
${\mathrm{By}}_{\max}$		−818.5	−805.8	−782.2	−693.9	−688.3	−681.8	−640.9	−623	−605	−589.3	−571.9
${\mathrm{By}}_{\min}$		−1996.3	−1956	−1834.9	−1592.5	−1508	−1452.5	−1382.2	−1362.2	−1350.5	−1342.6	−1338.7
T		0	2.8	15.6	39.6	53.7	56.1	61.5	63.8	65.3	65.9	65.2
$A_{T}$		7.0714	7.0477	6.9591	6.8008	6.7089	6.6473	6.6084	6.6056	6.6141	6.6245	6.6422

**Table A5:** Calculation result of $A_{T}$ for 20 mm diameter steel bar (LH = 1 cm).

	TLD	0	4	8	12	16	20	30	40	50	60	70	80	90
Parameter														
${\mathrm{By}}_{\max}$		−1003.9	−992.5	−992.5	−622	−519.9	−470.9	−349.4	−390.3	−309.6	−321.1	−331.8	−341.3	−346.5
${\mathrm{By}}_{\min}$		−1949.5	−1897.2	−1846.3	−1423.4	−1252.6	−1188.3	−1033.2	−1130.5	−1063.8	−1063.6	−1085.9	−1095.3	−1100.6
T		0	20	49.3	88.2	127.2	144.2	143.8	156.7	172.4	181.1	185.2	186.7	186.5
$A_{T}$		6.8518	6.8076	6.7497	6.6864	6.5967	6.5756	6.5277	6.6069	6.6257	6.6100	6.6255	6.6254	6.6255

**Table A6:** Calculation result of $A_{T}$ for 20 mm diameter steel bar (LH = 3 cm).

	TLD	0	4	8	12	16	20	30	40	50	60	70	80	90
Parameter														
${\mathrm{By}}_{\max}$		−1074.1	−1072.2	−975.6	−693.7	−602.7	−534.9	−455.2	−417	−394	−398.1	−401.6	−404.8	−405.8
${\mathrm{By}}_{\min}$		−1990.2	−1967.3	−1845.9	−1515.3	−1388.7	−1311.5	−1171	−1179.5	−1142.4	−1138.9	−1144	−1122.9	−1132.9
T		0	20	49.3	88.2	127.2	144.2	143.8	156.7	172.4	181.1	185.2	186.7	186.5
$A_{T}$		6.8201	6.7969	6.7688	6.7113	6.6670	6.6549	6.5734	6.6366	6.6179	6.6077	6.6099	6.5766	6.5891

**Table A7:** Calculation result of $A_{T}$ for 20 mm diameter steel bar (LH = 5 cm).

	TLD	0	4	8	12	16	20	30	40	50	60	70	80	90
Parameter														
${\mathrm{By}}_{\max}$		−1102.3	−1037.1	−1002.1	−757.7	−674.5	−602.9	−524.7	−480	−457.5	−398.1	−453.8	−452.7	−449.9
${\mathrm{By}}_{\min}$		−1991.1	−1907.5	−1829.7	−1550.3	−1420.7	−1333.1	−1295.4	−1291.8	−1254.6	−1250.4	−1244.3	−1227.1	−1237.3
T		0	20	49.3	88.2	127.2	144.2	143.8	156.7	172.4	181.1	185.2	186.7	186.5
$A_{T}$		6.7899	6.7690	6.7185	6.6753	6.6150	6.5933	6.6473	6.6993	6.6810	6.7479	6.6727	6.6521	6.6687

**Table A8:** Calculation result of $A_{T}$ for 20 mm diameter steel bar (LH = 7 cm).

	TLD	0	4	8	12	16	20	30	40	50	60	70	80	90
Parameter														
${\mathrm{By}}_{\max}$		−1099.4	−1082.6	−1014	−782.4	−738.9	−657.5	−571.6	−525.9	−503.3	−496.6	−492.3	−487.3	−483.2
${\mathrm{By}}_{\min}$		−1997.3	−1952.2	−1853.9	−1581.4	−1496.7	−1402.7	−1291.6	−1277.3	−1253.5	−1239.3	−1235.8	−1237.7	−1232.1
T		0	20	49.3	88.2	127.2	144.2	143.8	156.7	172.4	181.1	185.2	186.7	186.5
A_T_		6.8001	6.7680	6.7333	6.6834	6.6304	6.6137	6.5793	6.6219	6.6203	6.6103	6.6114	6.6206	6.6186

**Table A9:** Calculation result of A_T_ for 25 mm diameter steel bar (LH = 1 cm).

	TLD	0	4	8	12	16	20	30	40	50	60	70	80	90
Parameter														
By_max_		−1080.3	−975.6	−925.1	−635.5	−465.4	−404.4	−397.2	−279	−283.3	−288.9	−290.2	−295.4	−298.3
By_min_		−1961.3	−1852.7	−1721.1	−1376.7	−1109.5	−1005.9	−815.7	−647.3	−640.2	−642.3	−630.3	−613.2	−618.3
T		0	10.9	47.6	95	158.5	208.5	216.1	236.9	257	266.3	276.3	286.9	273.2
A_T_		6.7811	6.7766	6.6796	6.6083	6.4679	6.3994	6.0367	5.9089	5.8775	5.8676	5.8292	5.7614	5.7683

**Table A10:** Calculation result of A_T_ for 25 mm diameter steel bar (LH = 3 cm).

	TLD	0	4	8	12	16	20	30	40	50	60	70	80	90
Parameter														
By_max_		−1079.9	−1051.5	−988.6	−774.3	−587.7	−497.1	−476.6	−373.5	−370.3	−369	−385.7	−382.3	−400.8
By_min_		−1984.3	−1949.7	−1839.5	−1571.2	−1332.8	−1185.4	−1064.1	−1017	−1001.4	−993.4	−970.6	−955.4	−942.2
T		0	10.9	47.6	95	158.5	208.5	216.1	236.9	257	266.3	276.3	286.9	273.2
A_T_		6.8073	6.8004	6.7463	6.6807	6.6135	6.5342	6.3759	6.4669	6.4475	6.4368	6.3714	6.3511	6.2942

**Table A11:** Calculation result of A_T_ for 25 mm diameter steel bar (LH = 5 cm).

	TLD	0	4	8	12	16	20	30	40	50	60	70	80	90
Parameter														
By_max_		−1128.5	−1098.7	−1054.9	−832.5	−655.2	−564.3	−536.7	−444.8	−436.1	−428.5	−451.3	−440.2	−436.3
By_min_		−1921.7	−1885.4	−1806.2	−1544.3	−1314.9	−1181.2	−1088.5	−1026.8	−1012.7	−1002.1	−960.6	−965.6	−960.4
T		0	10.9	47.6	95	158.5	208.5	216.1	236.9	257	266.3	276.3	286.9	273.2
A_T_		6.6761	6.6678	6.6218	6.5678	6.4918	6.4247	6.3132	6.3665	6.3571	6.3519	6.2330	6.2642	6.2617

**Table A12:** Calculation result of A_T_ for 25 mm diameter steel bar (LH = 7 cm).

	TLD	0	4	8	12	16	20	30	40	50	60	70	80	90
Parameter														
By_max_		−1152.7	−1101.5	−1079.3	−872.3	−701.5	−611.9	−577.5	−495.5	−481.9	−472.9	−470.2	−471.3	−485.6
By_min_		−1974.8	−1914.8	−1856.5	−1614.7	−1387.9	−1258.7	−1171.8	−1108.5	−1090.9	−1083.2	−1053.2	−1052	−1050.8
T		0	10.9	47.6	95	158.5	208.5	216.1	236.9	257	266.3	276.3	286.9	273.2
A_T_		6.7119	6.7011	6.6557	6.6099	6.5315	6.4720	6.3874	6.4184	6.4118	6.4140	6.3682	6.3642	6.3372
